# The genome sequence of a seed weevil, *Aspidapion aeneum *(Fabricius, 1775)

**DOI:** 10.12688/wellcomeopenres.23613.1

**Published:** 2025-02-03

**Authors:** Maxwell V. L. Barclay, Toby Turner, Natural History Museum Genome Acquisition Lab, Darwin Tree of Life Barcoding collective, Wellcome Sanger Institute Tree of Life Management, Samples and Laboratory team, Wellcome Sanger Institute Scientific Operations: Sequencing Operations, Wellcome Sanger Institute Tree of Life Core Informatics team, Tree of Life Core Informatics collective, Darwin Tree of Life Consortium

**Affiliations:** 1 Natural History Museum, London, England, UK

**Keywords:** Aspidapion aeneum, seed weevil, genome sequence, chromosomal, Coleoptera

## Abstract

We present a genome assembly from a specimen of *Aspidapion aeneum* (seed weevil; Arthropoda; Insecta; Coleoptera; Apionidae). The genome sequence has a total length of 1,286.20 megabases. Most of the assembly (98.78%) is scaffolded into 11 chromosomal pseudomolecules. The mitochondrial genome has also been assembled and is 21.49 kilobases in length.

## Species taxonomy

Eukaryota; Opisthokonta; Metazoa; Eumetazoa; Bilateria; Protostomia; Ecdysozoa; Panarthropoda; Arthropoda; Mandibulata; Pancrustacea; Hexapoda; Insecta; Dicondylia; Pterygota; Neoptera; Endopterygota; Coleoptera; Polyphaga; Cucujiformia; Curculionoidea; Apionidae; *Aspidapion*; *Aspidapion aeneum* (Fabricius, 1775) (NCBI:txid877730)

## Background

The family Apionidae Schoenherr, 1823, commonly known as seed weevils, is a large family of weevils (Curculionoidea), most of which develop inside living plants (though, despite the common name, not always in the seeds) and show a high degree of host-plant specificity. They are mostly small, dark-coloured weevils with straight antennae (not angled or geniculate as in the related family Curculionidae). There are 87 species reported from Britain and Ireland ( Duff, 2018), including three members of the genus *Aspidapion*.

All species of *Aspidapion* in Britain are associated with mallows and their relatives (family Malvaceae) ( Morris, 1990). *A. aeneum* is oligophagous on Malvaceae, commonly found in Britain on *Malva sylvestris*, where larvae feed within the stems of the plant ( Duff, 2016). Across its range, it can be found on several genera and species of Malvaceae, including *Malva moschata*, *M. pusilla*, *M. neglecta*, *M. sylvestris*, *Alcea rosea*, *Althaea officinalis*, and *Lavatera thuringiaca* ( Yunakov *et al.*, 2018).

At 2.9–3.6 mm long, *A. aeneum* is the largest member of its genus and one of the largest Apionidae occurring in Britain. It can be identified using Morris (1990) and Duff (2016) and is recognised by its large size, metallic elytra with bronzy or coppery lustre, occasionally violet or bluish, contrasting with generally non-metallic foreparts, a deep longitudinal impression on the frons, scutellum simple or with very small basal keels; elytra with metallic bronzy or coppery lustre, occasionally violet or bluish, with smooth intervals appearing five times as wide as the striae ( Duff, 2016). It has been classified in the subgenus *Koestlinia*, Alonso-Zarazaga. In many recent works, the family Apionidae is treated as a subfamily of Brentidae (e.g. Alonso-Zarazaga *et al.*, 2017).

*Aspidapion aeneum* (Fabricius, 1775) ranges throughout the Palaearctic, being present in Europe, North Africa, and Western Asia, with Alonso-Zarazaga *et al*. (2017) listing the following countries: Albania, Austria, Bulgaria, Croatia, Russia (central territories), Czech Republic, Denmark, France, Great Britain, Germany, Greece (including Crete), Hungary, Italy, Luxembourg, Malta, Macedonia, Netherlands, Poland, Portugal, Serbia, Slovakia, Spain, Russia (southern European territories), Sweden, Switzerland, Ukraine, Algeria, Morocco, Tunisia, Azerbaijan, Afghanistan, Armenia, Cyprus, Georgia, Iran, Israel, Kyrgyzstan, Kazakhstan, Lebanon, Syria, Turkmenistan, and Uzbekistan. The beetle also occurs in Ireland, which was omitted from this list ( Morris, 1990). In England, it is widespread in central and southeastern regions and more local in Wales ( Duff, 2016). It can be found in or around meadows, arable/agricultural land, and ruderal early succession habitats. Towards the north and west of its range in Britain, for example in Wales and the Isle of Man, it tends to be more coastal.

**Figure 1.**
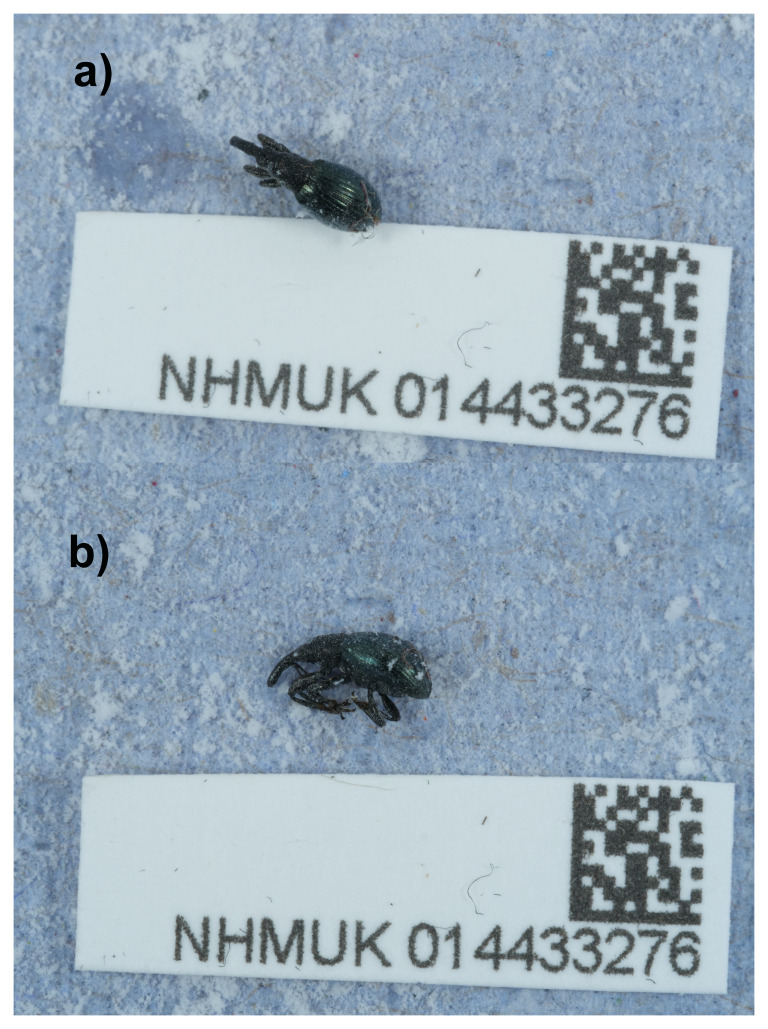
Photographs of the *Aspidapion aeneum* specimen used for genome sequencing.

The genome of the weevil, *Aspidapion aeneum* ( Figure 1), was sequenced as part of the Darwin Tree of Life Project, a collaborative effort to sequence all named eukaryotic species in the Atlantic Archipelago of Britain and Ireland. Here we present a chromosomal-level genome sequence for *Aspidapion aeneum*, based on a specimen from Aldeburgh, England, United Kingdom. The specimen used for sequencing was collected and identified by M.V.L. Barclay, by sweeping coastal ruderal vegetation at Aldeburgh, Suffolk, England, on 6 June 2021.

## Genome sequence report

The genome of *Aspidapion aeneum* was sequenced using Pacific Biosciences single-molecule HiFi long reads, generating a total of 28.25 Gb (gigabases) from 2.36 million reads, providing an estimated 19-fold coverage. Primary assembly contigs were scaffolded with chromosome conformation Hi-C data, which produced 99.49 Gb from 658.86 million reads. Specimen and sequencing details are summarised in Table 1.

Assembly errors were corrected by manual curation, including 12 missing joins or mis-joins and four haplotypic duplications. This reduced the assembly length by 0.77% and the scaffold number by 0.66%, and also decreased the scaffold N50 by 5.27%. The final assembly has a total length of 1,286.20 Mb in 150 sequence scaffolds, with 631 gaps, and a scaffold N50 of 125.8 Mb ( Table 2).

**Figure 2.**
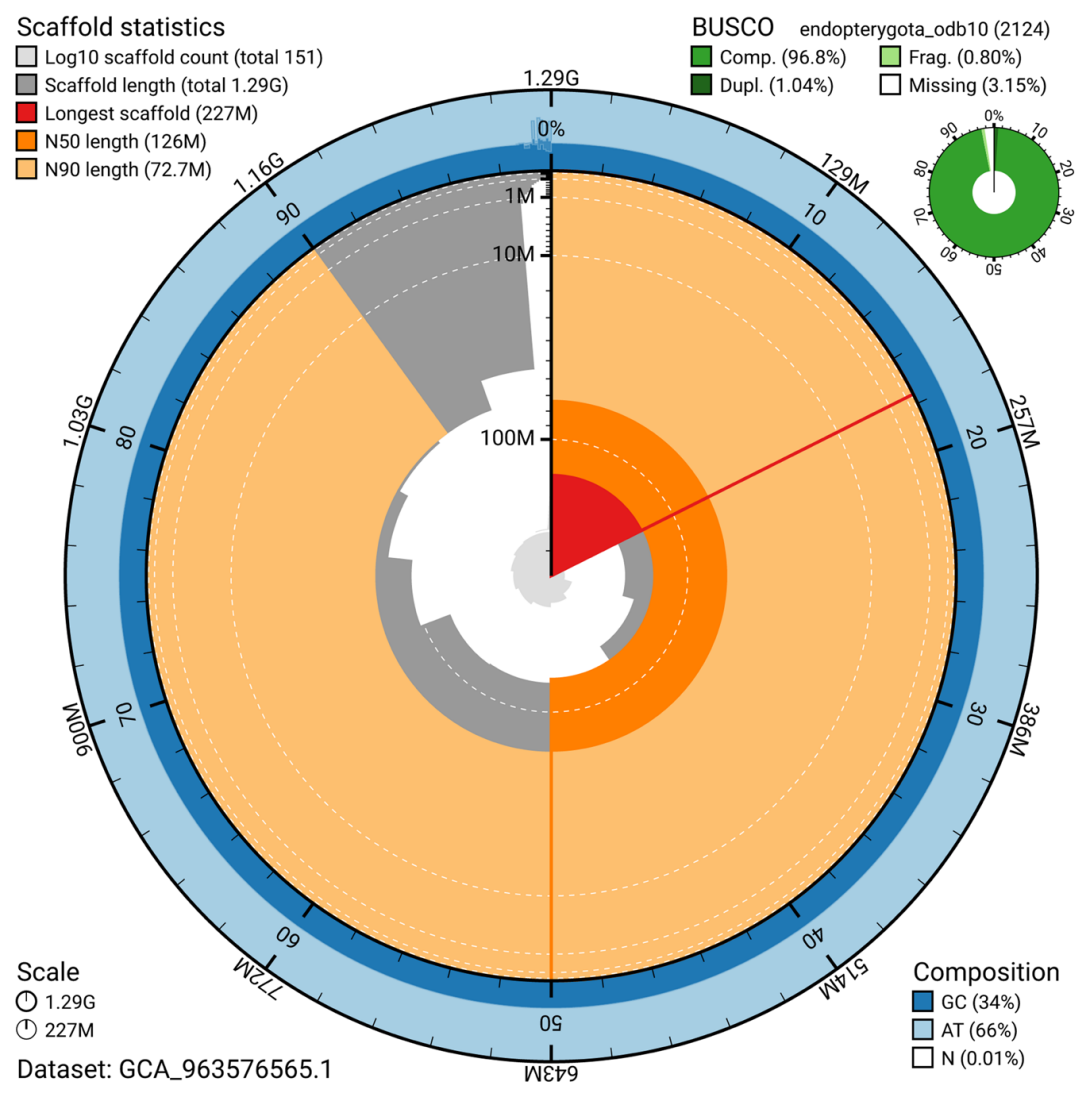
Genome assembly of *Aspidapion aeneum*, icAspAene1.1: metrics. The BlobToolKit snail plot provides an overview of assembly metrics and BUSCO gene completeness. The circumference represents the length of the whole genome sequence, and the main plot is divided into 1,000 bins around the circumference. The outermost blue tracks display the distribution of GC, AT, and N percentages across the bins. Scaffolds are arranged clockwise from longest to shortest and are depicted in dark grey. The longest scaffold is indicated by the red arc, and the deeper orange and pale orange arcs represent the N50 and N90 lengths. A light grey spiral at the centre shows the cumulative scaffold count on a logarithmic scale. A summary of complete, fragmented, duplicated, and missing BUSCO genes in the endopterygota_odb10 set is presented at the top right. An interactive version of this figure is available at https://blobtoolkit.genomehubs.org/view/GCA_963576565.1/dataset/GCA_963576565.1/snail.

**Figure 3.**
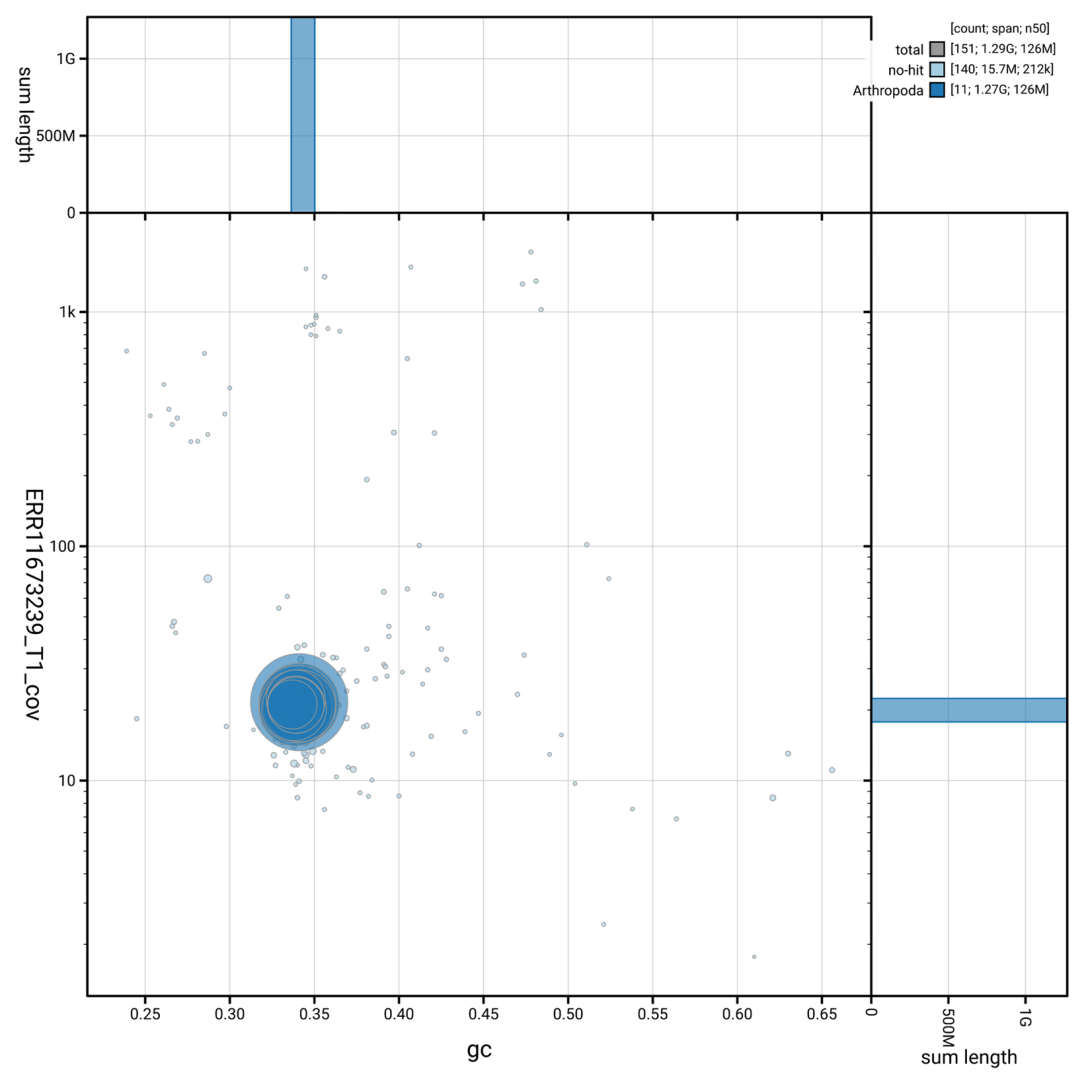
Genome assembly of *Aspidapion aeneum*, icAspAene1.1: BlobToolKit GC-coverage plot showing sequence coverage (vertical axis) and GC content (horizontal axis). The circles represent scaffolds, with the size proportional to scaffold length and the colour representing phylum membership. The histograms along the axes display the total length of sequences distributed across different levels of coverage and GC content. An interactive version of this figure is available at https://blobtoolkit.genomehubs.org/view/GCA_963576565.1/dataset/GCA_963576565.1/blob.

**Figure 4.**
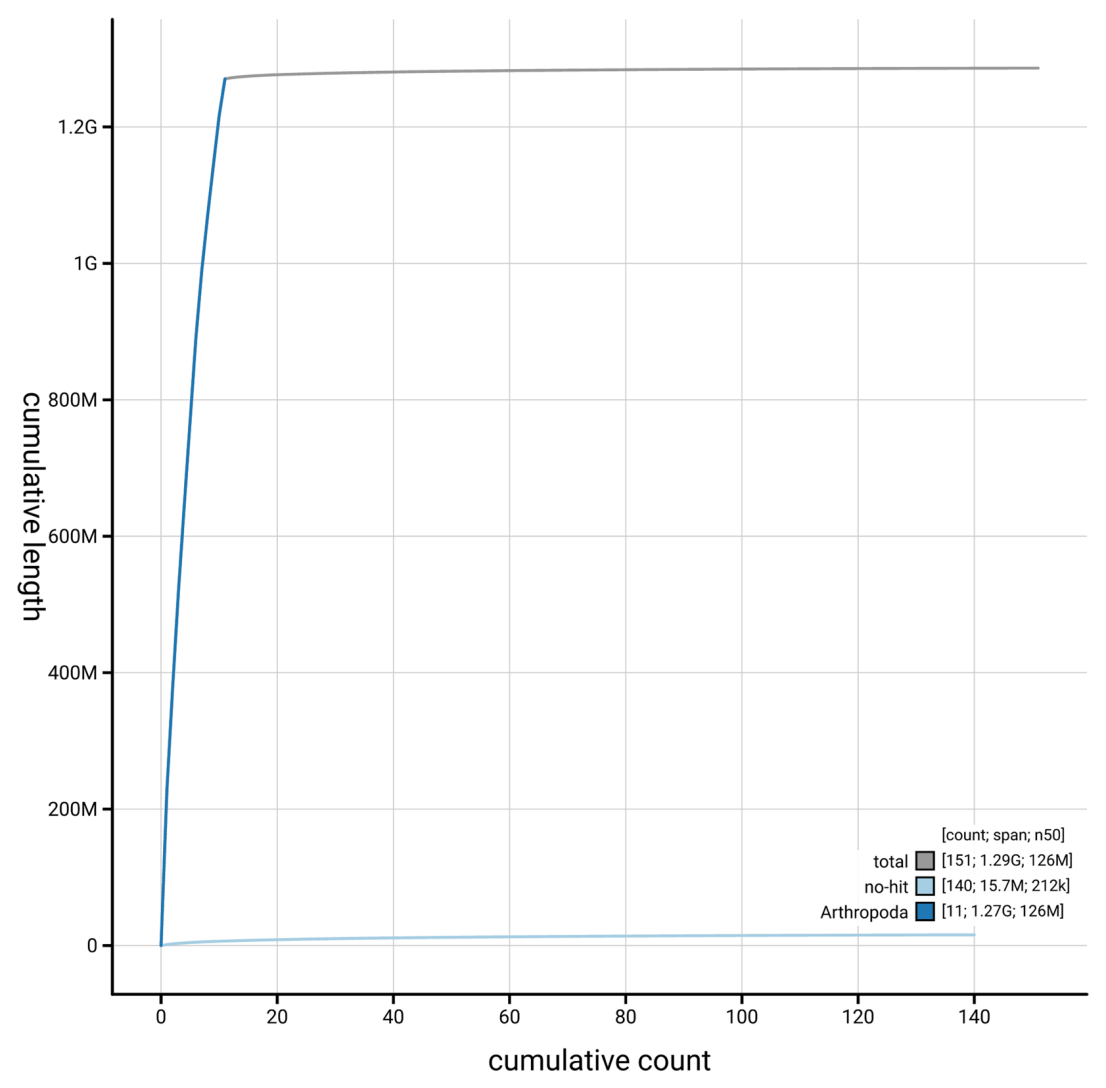
Genome assembly of *Aspidapion aeneum* icAspAene1.1: BlobToolKit cumulative sequence plot. The grey line shows cumulative length for all scaffolds. Coloured lines show cumulative lengths of scaffolds assigned to each phylum using the buscogenes taxrule. An interactive version of this figure is available at https://blobtoolkit.genomehubs.org/view/GCA_963576565.1/dataset/GCA_963576565.1/cumulative.

The snail plot in Figure 2 provides a summary of the assembly statistics, indicating the distribution of scaffold lengths and other assembly metrics. Figure 3 shows the distribution of scaffolds by GC proportion and coverage. Figure 4 presents a cumulative assembly plot, with separate curves representing different scaffold subsets assigned to various phyla, illustrating the completeness of the assembly.

**Figure 5.**
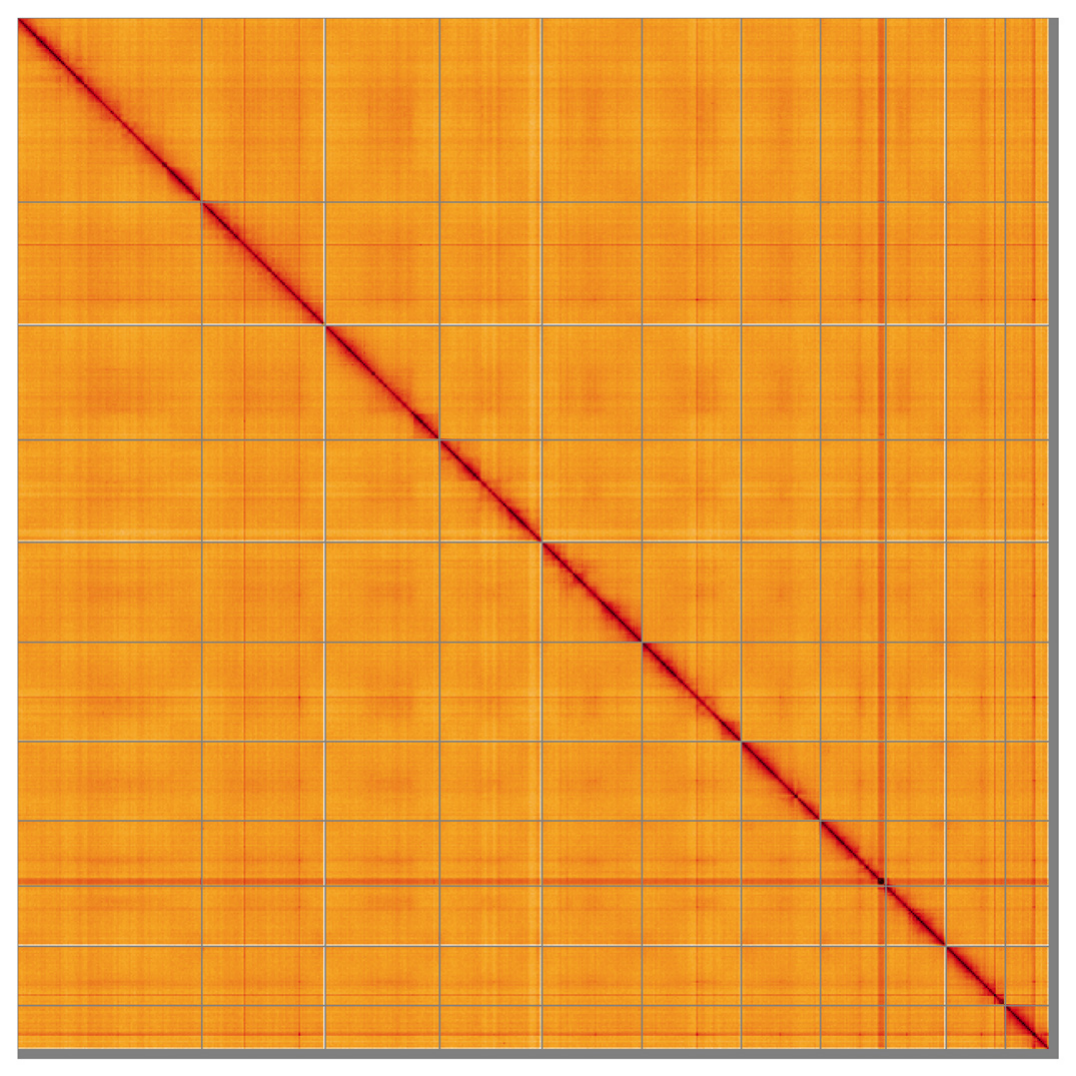
Genome assembly of *Aspidapion aeneum* icAspAene1.1: Hi-C contact map of the icAspAene1.1 assembly, visualised using HiGlass. Chromosomes are shown in order of size from left to right and top to bottom. An interactive version of this figure may be viewed at https://www.flickr.com/photos/84259756@N05/49838397711.

Most of the assembly sequence (98.78%) was assigned to 11 chromosomal-level scaffolds. These chromosome-level scaffolds, confirmed by the Hi-C data, are named in order of size ( Figure 5; Table 3). No sex chromosome could be identified, but the read coverage suggests that the specimen is the homogametic sex.

**Table 1.** Specimen and sequencing data for *Aspidapion aeneum*.

Project information			
**Study title**	Aspidapion aeneum		
**Umbrella BioProject**	PRJEB64084		
**Species**	*Aspidapion aeneum*		
**BioSample**	SAMEA111458458		
**NCBI taxonomy ID**	877730		
Specimen information			
**Technology**	**ToLID**	**BioSample accession**	**Organism part**
**PacBio long read** **sequencing**	icAspAene1	SAMEA111458555	Thorax and leg
**Hi-C sequencing**	icAspAene1	SAMEA111458553	Head
Sequencing information			
**Platform**	**Run accession**	**Read count**	**Base count (Gb)**
**Hi-C Illumina NovaSeq** **6000**	ERR11679404	6.59e+08	99.49
**PacBio Sequel IIe**	ERR11673239	2.36e+06	28.25

**Table 2.** Genome assembly data for *Aspidapion aeneum*, icAspAene1.1.

Genome assembly		
Assembly name	icAspAene1.1	
Assembly accession	GCA_963576565.1	
*Accession of alternate haplotype*	*GCA_963576425.1*	
Span (Mb)	1,286.20	
Number of contigs	782	
Number of scaffolds	150	
Longest scaffold (Mb)	227.08	
Assembly metrics *		*Benchmark*
Contig N50 length (Mb)	3.5	*≥ 1 Mb*
Scaffold N50 length (Mb)	125.8	*= chromosome N50*
Consensus quality (QV)	56.5	*≥ 40*
*k*-mer completeness	Primary: 74.33%; alternate: 73.15%; combined: 96.14%	*≥ 95%*
BUSCO v5.4.3 lineage: endopterygota_odb10	C:96.6%[S:95.6%,D:1.0%], F:0.9%,M:2.5%,n:2,124	*S > 90%*, *D < 5%*
Percentage of assembly mapped to chromosomes	98.78%	*≥ 90%*
Sex chromosomes	Not identified	*localised homologous* *pairs*
Organelles	Mitochondrial genome: 21.49 kb	*complete single alleles*

* Assembly metric benchmarks are adapted from Rhie *et al.* (2021) and the Earth BioGenome Project Report on Assembly Standards September 2024. ** BUSCO scores based on the endopterygota_odb10 BUSCO set using version 5.4.3. C = complete [S = single copy, D = duplicated], F = fragmented, M = missing, n = number of orthologues in comparison. A full set of BUSCO scores is available at https://blobtoolkit.genomehubs.org/view/Aspidapion_aeneum/dataset/GCA_963576565.1/busco

**Table 3.** Chromosomal pseudomolecules in the genome assembly of *Aspidapion aeneum*, icAspAene1.

INSDC accession	Name	Length (Mb)	GC%
OY755038.1	1	227.08	34.0
OY755039.1	2	151.81	34.0
OY755040.1	3	141.12	34.0
OY755041.1	4	125.84	34.0
OY755042.1	5	122.99	34.0
OY755043.1	6	122.19	34.0
OY755044.1	7	97.51	34.0
OY755045.1	8	80.5	34.0
OY755046.1	9	74.44	34.0
OY755047.1	10	72.7	34.0
OY755048.1	11	54.33	33.5
OY755049.1	MT	0.02	24.0

While not fully phased, the assembly deposited is of one haplotype. Contigs corresponding to an alternate haplotype have also been deposited. The mitochondrial genome was also assembled and can be found as a contig within the multifasta file of the genome submission, and as a separate fasta file with accession OY755049.1.

The final assembly has a Quality Value (QV) of 56.5. The *k*-mer completeness was estimated as 74.33% for the primary assembly, 73.15% for the alternate haplotype, and 96.14% for the combined assemblies. BUSCO (v5.4.3) analysis using the endopterygota_odb10 reference set ( *n* = 2,124) indicated a completeness score of 96.6% (single = 95.6%, duplicated = 1.0%). The assembly achieves the EBP reference standard of 6.C.57. Other quality metrics are given in Table 2. 

## Methods

### Sample acquisition and DNA barcoding

A specimen of *Aspidapion aeneum* (specimen ID NHMUK014433276, ToLID icAspAene1) was collected from Aldeburgh, England, United Kingdom (latitude 52.16, longitude 1.60) on 2021-06-06. The specimen was collected and identified by Maxwell Barclay (Natural History Museum) and preserved by dry freezing at –80 °C.

The initial identification was verified by an additional DNA barcoding process according to the framework developed by Twyford *et al*. (2024). A small sample was dissected from the specimens and stored in ethanol, while the remaining parts were shipped on dry ice to the Wellcome Sanger Institute (WSI). The tissue was lysed, the COI marker region was amplified by PCR, and amplicons were sequenced and compared to the BOLD database, confirming the species identification ( Crowley *et al.*, 2023). Following whole genome sequence generation, the relevant DNA barcode region was also used alongside the initial barcoding data for sample tracking at the WSI ( Twyford *et al.*, 2024). The standard operating procedures for Darwin Tree of Life barcoding have been deposited on protocols.io ( Beasley *et al.*, 2023).

### Nucleic acid extraction

The workflow for high molecular weight (HMW) DNA extraction at the Wellcome Sanger Institute (WSI) Tree of Life Core Laboratory includes a sequence of procedures: sample preparation and homogenisation, DNA extraction, fragmentation and purification. Detailed protocols are available on protocols.io ( Denton *et al.*, 2023b). The icAspAene1 sample was prepared for DNA extraction by weighing and dissecting it on dry ice ( Jay *et al.*, 2023). Tissue from the thorax and leg was homogenised using a PowerMasher II tissue disruptor ( Denton *et al.*, 2023a).

HMW DNA was extracted in the WSI Scientific Operations core using the Automated MagAttract v2 protocol ( Oatley *et al.*, 2023). The DNA was sheared into an average fragment size of 12–20 kb in a Megaruptor 3 system ( Bates *et al.*, 2023). Sheared DNA was purified by solid-phase reversible immobilisation, using AMPure PB beads to eliminate shorter fragments and concentrate the DNA ( Strickland *et al.*, 2023). The concentration of the sheared and purified DNA was assessed using a Nanodrop spectrophotometer and Qubit Fluorometer using the Qubit dsDNA High Sensitivity Assay kit. Fragment size distribution was evaluated by running the sample on the FemtoPulse system.

### Hi-C sample preparation

Tissue from the head of the icAspAene1 sample was processed at the WSI Scientific Operations core, using the Arima-HiC v2 kit. Tissue (stored at –80 °C) was fixed, and the DNA crosslinked using a TC buffer with 22% formaldehyde. After crosslinking, the tissue was homogenised using the Diagnocine Power Masher-II and BioMasher-II tubes and pestles. Following the kit manufacturer's instructions, crosslinked DNA was digested using a restriction enzyme master mix. The 5’-overhangs were then filled in and labelled with biotinylated nucleotides and proximally ligated. An overnight incubation was carried out for enzymes to digest remaining proteins and for crosslinks to reverse. A clean up was performed with SPRIselect beads prior to library preparation.

### Library preparation and sequencing

***PacBio Hifi***

Libraries were prepared using the PacBio Express Template Preparation Kit v2.0 (Pacific Biosciences, California, USA) as per the manufacturer's instructions. The kit includes the reagents required for removal of single-strand overhangs, DNA damage repair, end repair/A-tailing, adapter ligation, and nuclease treatment. Library preparation also included a library purification step using AMPure PB beads (Pacific Biosciences, California, USA) and size selection step to remove templates shorter than 3 kb using AMPure PB modified SPRI. DNA concentration was quantified using the Qubit Fluorometer v2.0 (Thermo Fisher Scientific) and Qubit HS Assay Kit and the final library fragment size analysis was carried out using the Agilent Femto Pulse Automated Pulsed Field CE Instrument (Agilent Technologies).

Samples were sequenced using the Sequel IIe system (Pacific Biosciences, California, USA). The concentration of the library loaded onto the Sequel IIe was in the range 40–135 pM. The SMRT link software, a PacBio web-based end-to-end workflow manager, was used to set-up and monitor the run, as well as perform primary and secondary analysis of the data upon completion.

***Hi-C***

For Hi-C library preparation, DNA was fragmented to a size of 400 to 600 bp using a Covaris E220 sonicator. The DNA was then enriched, barcoded, and amplified using the NEBNext Ultra II DNA Library Prep Kit following manufacturers’ instructions. The Hi-C sequencing was performed using paired-end sequencing with a read length of 150 bp on an Illumina NovaSeq 6000 instrument.

### Genome assembly, curation and evaluation

***Assembly***

The HiFi reads were first assembled using Hifiasm ( Cheng *et al.*, 2021) with the --primary option. Haplotypic duplications were identified and removed using purge_dups ( Guan *et al.*, 2020). The Hi-C reads were mapped to the primary contigs using bwa-mem2 ( Vasimuddin *et al.*, 2019). The contigs were further scaffolded using the provided Hi-C data ( Rao *et al.*, 2014) in YaHS ( Zhou *et al.*, 2023) using the --break option for handling potential misassemblies. The scaffolded assemblies were evaluated using Gfastats ( Formenti *et al.*, 2022), BUSCO ( Manni *et al.*, 2021) and MERQURY.FK ( Rhie *et al.*, 2020).

The mitochondrial genome was assembled using MitoHiFi ( Uliano-Silva *et al.*, 2023), which runs MitoFinder ( Allio *et al.*, 2020) and uses these annotations to select the final mitochondrial contig and to ensure the general quality of the sequence.

***Assembly curation***

The assembly was decontaminated using the Assembly Screen for Cobionts and Contaminants (ASCC) pipeline (article in preparation). Flat files and maps used in curation were generated in TreeVal ( Pointon *et al.*, 2023). Manual curation was primarily conducted using PretextView ( Harry, 2022), with additional insights provided by JBrowse2 ( Diesh *et al.*, 2023) and HiGlass ( Kerpedjiev *et al.*, 2018). Scaffolds were visually inspected and corrected as described by Howe *et al*. (2021). Any identified contamination, missed joins, and mis-joins were corrected, and duplicate sequences were tagged and removed. The curation process is documented at https://gitlab.com/wtsi-grit/rapid-curation (article in preparation).

***Assembly quality assessment***

The Merqury.FK tool ( Rhie *et al.*, 2020) was used to evaluate *k*-mer completeness and assembly quality for the primary and alternate haplotypes using the *k*-mer databases ( *k* = 31) that were pre-computed prior to genome assembly. The analysis outputs included assembly QV scores and completeness statistics.

A Hi-C contact map was produced for the final, public version of the assembly. The Hi-C reads were aligned using bwa-mem2 ( Vasimuddin *et al.*, 2019) and the alignment files were combined using SAMtools ( Danecek *et al.*, 2021). The Hi-C alignments were converted into a contact map using BEDTools ( Quinlan & Hall, 2010) and the Cooler tool suite ( Abdennur & Mirny, 2020). The contact map is visualised in HiGlass ( Kerpedjiev *et al.*, 2018).

The blobtoolkit pipeline is a Nextflow port of the previous Snakemake Blobtoolkit pipeline ( Challis *et al.*, 2020). It aligns the PacBio reads in SAMtools and minimap2 ( Li, 2018) and generates coverage tracks for regions of fixed size. In parallel, it queries the GoaT database ( Challis *et al.*, 2023) to identify all matching BUSCO lineages to run BUSCO ( Manni *et al.*, 2021). For the three domain-level BUSCO lineages, the pipeline aligns the BUSCO genes to the UniProt Reference Proteomes database ( Bateman *et al.*, 2023) with DIAMOND blastp ( Buchfink *et al.*, 2021). The genome is also divided into chunks according to the density of the BUSCO genes from the closest taxonomic lineage, and each chunk is aligned to the UniProt Reference Proteomes database using DIAMOND blastx. Genome sequences without a hit are chunked using seqtk and aligned to the NT database with blastn ( Altschul *et al.*, 1990). The blobtools suite combines all these outputs into a blobdir for visualisation.

The blobtoolkit pipeline was developed using nf-core tooling ( Ewels *et al.*, 2020) and MultiQC ( Ewels *et al.*, 2016), relying on the Conda package manager, the Bioconda initiative ( Grüning *et al.*, 2018), the Biocontainers infrastructure ( da Veiga Leprevost *et al.*, 2017), as well as the Docker ( Merkel, 2014) and Singularity ( Kurtzer *et al.*, 2017) containerisation solutions.

Table 4 contains a list of relevant software tool versions and sources.

**Table 4.** Software tools: versions and sources.

Software tool	Version	Source
BEDTools	2.30.0	https://github.com/arq5x/bedtools2
BLAST	2.14.0	http://ftp.ncbi.nlm.nih.gov/blast/executables/blast+/
BlobToolKit	4.3.7	https://github.com/blobtoolkit/blobtoolkit
BUSCO	5.4.3 and 5.5.0	https://gitlab.com/ezlab/busco
bwa-mem2	2.2.1	https://github.com/bwa-mem2/bwa-mem2
Cooler	0.8.11	https://github.com/open2c/cooler
DIAMOND	2.1.8	https://github.com/bbuchfink/diamond
fasta_windows	0.2.4	https://github.com/tolkit/fasta_windows
FastK	427104ea91c78c3b8b8b49f1a 7d6bbeaa869ba1c	https://github.com/thegenemyers/FASTK
Gfastats	1.3.6	https://github.com/vgl-hub/gfastats
GoaT CLI	0.2.5	https://github.com/genomehubs/goat-cli
Hifiasm	0.19.8-r587	https://github.com/chhylp123/hifiasm
HiGlass	44086069ee7d4d3f6f3f001256 9789ec138f42b84aa44357826 c0b6753eb28de	https://github.com/higlass/higlass
Merqury.FK	d00d98157618f4e8d1a919002 6b19b471055b22e	https://github.com/thegenemyers/MERQURY.FK
MitoHiFi	3	https://github.com/marcelauliano/MitoHiFi
MultiQC	1.14, 1.17, and 1.18	https://github.com/MultiQC/MultiQC
NCBI Datasets	15.12.0	https://github.com/ncbi/datasets
Nextflow	23.04.0-5857	https://github.com/nextflow-io/nextflow
PretextView	0.2.5	https://github.com/sanger-tol/PretextView
purge_dups	1.2.5	https://github.com/dfguan/purge_dups
samtools	1.16.1, 1.17, and 1.18	https://github.com/samtools/samtools
sanger-tol/ascc	-	https://github.com/sanger-tol/ascc
sanger-tol/ genomenote	1.1.1	https://github.com/sanger-tol/genomenote
sanger-tol/ readmapping	1.2.1	https://github.com/sanger-tol/readmapping
Seqtk	1.3	https://github.com/lh3/seqtk
Singularity	3.9.0	https://github.com/sylabs/singularity
TreeVal	1.0.0	https://github.com/sanger-tol/treeval
YaHS	1.2a.2	https://github.com/c-zhou/yahs

### Wellcome Sanger Institute – Legal and Governance

The materials that have contributed to this genome note have been supplied by a Darwin Tree of Life Partner. The submission of materials by a Darwin Tree of Life Partner is subject to the **‘Darwin Tree of Life Project Sampling Code of Practice’**, which can be found in full on the Darwin Tree of Life website here. By agreeing with and signing up to the Sampling Code of Practice, the Darwin Tree of Life Partner agrees they will meet the legal and ethical requirements and standards set out within this document in respect of all samples acquired for, and supplied to, the Darwin Tree of Life Project.

Further, the Wellcome Sanger Institute employs a process whereby due diligence is carried out proportionate to the nature of the materials themselves, and the circumstances under which they have been/are to be collected and provided for use. The purpose of this is to address and mitigate any potential legal and/or ethical implications of receipt and use of the materials as part of the research project, and to ensure that in doing so we align with best practice wherever possible. The overarching areas of consideration are:

•   Ethical review of provenance and sourcing of the material

•   Legality of collection, transfer and use (national and international)

Each transfer of samples is further undertaken according to a Research Collaboration Agreement or Material Transfer Agreement entered into by the Darwin Tree of Life Partner, Genome Research Limited (operating as the Wellcome Sanger Institute), and in some circumstances other Darwin Tree of Life collaborators.

## Data Availability

European Nucleotide Archive: Aspidapion aeneum. Accession number PRJEB64084; https://identifiers.org/ena.embl/PRJEB64084. The genome sequence is released openly for reuse. The *Aspidapion aeneum* genome sequencing initiative is part of the Darwin Tree of Life (DToL) project. All raw sequence data and the assembly have been deposited in INSDC databases. The genome will be annotated using available RNA-Seq data and presented through the Ensembl pipeline at the European Bioinformatics Institute. Raw data and assembly accession identifiers are reported in Table 1 and Table 2.

## Author information

Members of the Natural History Museum Genome Acquisition Lab are listed here: https://doi.org/10.5281/zenodo.12159242.

Members of the Darwin Tree of Life Barcoding collective are listed here: https://doi.org/10.5281/zenodo.12158331.

Members of the Wellcome Sanger Institute Tree of Life Management, Samples and Laboratory team are listed here: https://doi.org/10.5281/zenodo.12162482.

Members of Wellcome Sanger Institute Scientific Operations: Sequencing Operations are listed here: https://doi.org/10.5281/zenodo.12165051.

Members of the Wellcome Sanger Institute Tree of Life Core Informatics team are listed here: https://doi.org/10.5281/zenodo.12160324.

Members of the Tree of Life Core Informatics collective are listed here: https://doi.org/10.5281/zenodo.12205391.

Members of the Darwin Tree of Life Consortium are listed here: https://doi.org/10.5281/zenodo.4783558.
