# Supplementary material for: The genome sequence of a seed weevil, Aspidapion aeneum (Fabricius, 1775)
Source: Wellcome Open Res. 2025 Feb 3;10:41. [Version 1] doi: 10.12688/wellcomeopenres.23613.1 (PMC11914875; doi:10.12688/wellcomeopenres.23613.1)
Supplement: Peer review history

# Reviewer report: Reviewer response for version 1

**DOI:** 10.21956/wellcomeopenres.26049.r118946

**Published:** 2025-03-28

**Authors:** Lapo Ragionieri

**Affiliations:** 1 Free University of Bolzano, Bolzano, Italy

DATA NOTE Review

 The genome sequence of a seed weevil, *Aspidapion aeneum* (Fabricius, 1775), authored by Maxwell Barclay and Toby Turner, details the sequencing and genome assembly of *Aspidapion aeneum*. The authors employed long-read sequencing technology (PacBio) to generate an initial assembly, which was then scaffolded using Hi-C, resulting in a chromosome-level genome assembly. Nearly all scaffolds were assigned to chromosomes, and the assembly also includes the mitochondrial genome. The genome assembly spans a total length of 1,286.20 Mb across 11 chromosomes.

 While the introduction and presentation of the results are well-executed, the authors did not provide any information regarding the masking of the genome. Below are specific comments and suggestions:

**Keywords**: To be effective, keywords should differ from the title.

**Chromosomes**: The identification of 11 chromosomes suggests that the specimen is of the homogametic sex. However, there is no information on whether the sample was male or female. In the methods section (Sample Acquisition and DNA Barcoding), the authors should indicate if the sex of the sample was verified before proceeding with genome extraction.

**Annotation**: The authors should identify and quantify transposable elements within the genome and include this annotation in the current data note.

**Command Lines**: It is standard practice to provide the command lines used for all software applications. Including this information would greatly benefit readers.

Are sufficient details of methods and materials provided to allow replication by others?

Partly

Is the rationale for creating the dataset(s) clearly described?

Yes

Are the datasets clearly presented in a useable and accessible format?

Yes

Are the protocols appropriate and is the work technically sound?

Yes

Reviewer Expertise:

genomic, transcriptomic, and proteomic

I confirm that I have read this submission and believe that I have an appropriate level of expertise to confirm that it is of an acceptable scientific standard.

# Reviewer report: Reviewer response for version 1

**DOI:** 10.21956/wellcomeopenres.26049.r118942

**Published:** 2025-03-17

**Authors:** Brent Emerson

**Affiliations:** 1 Institute of Natural Products and Agrobiology, La Laguna, Spain

In the first paragraph of the section “Genome sequence report”, it would be useful to include the genome size, to provide context for the other metrics that are mentioned.

 I’m not familiar genome assembly an annotation, but it surprised me that with a genome of this quality it is not possible to identify the sex chromosome. Why is that? Maybe some explanatory text around this point would be useful.

 The only real concern I have, and it is relatively minor, is that  the ASCC pipeline is in preparation. I have mentioned this in a previous review, and my concern is that I do not think that this complies with FAIR principles. Is the ASCC paper, or a version of it, now available on a pre-publication archive? As I say, this is a small concern, as it seems clear that this paper will be forthcoming. Perhaps at least identify who the authors of this upcoming paper are?

Are sufficient details of methods and materials provided to allow replication by others?

Partly

Is the rationale for creating the dataset(s) clearly described?

Yes

Are the datasets clearly presented in a useable and accessible format?

Yes

Are the protocols appropriate and is the work technically sound?

Yes

Reviewer Expertise:

Population genetics, phylogenetics, invertebrates

I confirm that I have read this submission and believe that I have an appropriate level of expertise to confirm that it is of an acceptable scientific standard.

# Reviewer report: Reviewer response for version 1

**DOI:** 10.21956/wellcomeopenres.26049.r118941

**Published:** 2025-03-11

**Authors:** Bryan Brunet

**Affiliations:** 1 Ottawa Research and Development Centre, Ontario, Canada

The manuscript entitled “The genome sequence of a seed weevil, *Aspidapion aeneum* (Fabricius, 1775)” by authors Barclay, Turner *et al.* presents a chromosome length genome assembly for the seed weevil species, *Aspidapion aeneum*, as well as a complete mitochondrial genome assembly. The authors use a combination of PacBio HiFi long reads and Hi-C data to assemble and scaffold the genome into 11 scaffolds that are 54-227 Mb in length. Assembly metrics are very good, and there is no doubt that the assembly is of high integrity. The methods are described in thorough detail and most, with exception to the ASCC pipeline, are already publicly available. Genome annotation has not been completed, but is planned, and the identities of the sex chromosomes remain unknown. The authors might consider conducting a synteny analysis with BUSCO orthologs using a tool such as ChromSyn ( https://github.com/slimsuite/chromsyn), in place of a fully annotated genome to attempt to identify the sex chromosomes. Only one minor edit is requested, and that is to report the currently accepted taxonomy for the species, rather than the outdated taxonomy that is used by the NCBI Taxonomy database. As already alluded to in the manuscript, Apionidae is now a subfamily under Brentidae.

Are sufficient details of methods and materials provided to allow replication by others?

Yes

Is the rationale for creating the dataset(s) clearly described?

Yes

Are the datasets clearly presented in a useable and accessible format?

Yes

Are the protocols appropriate and is the work technically sound?

Yes

Reviewer Expertise:

I am a research scientist with expertise in Hemiptera systematics and taxonomy.

I confirm that I have read this submission and believe that I have an appropriate level of expertise to confirm that it is of an acceptable scientific standard.

## References

[1] slimsuite/chromsyn. *GitHub* . Reference source
